# *Emergomyces pasteurianus* in Man Returning to the United States from Liberia and Review of the Literature

**DOI:** 10.3201/eid2903.221683

**Published:** 2023-03

**Authors:** Jacob Pierce, Sadia Sayeed, Christopher D. Doern, Alexandra L. Bryson

**Affiliations:** Virginia Commonwealth University Medical Center, Richmond, Virginia, USA

**Keywords:** Emergomyces pasteurianus, Emergomyces, fungi, fungal infections, respiratory infections, dimorphic fungus, Liberia, United States, Emmonsia pasteuriana

## Abstract

A 65-year-old man with HIV sought treatment for fever, weight loss, and productive cough after returning to the United States from Liberia. Fungal cultures grew *Emergomyces pasteurianus*, and the patient’s health improved after beginning voriconazole. We describe the clinical case and review the literature, treatment, and susceptibilities for *E. pasteurianus.*

In March 2019, a 65-year-old man sought treatment at an emergency department in Virginia, USA, for fever, odynophagia, weight loss, and productive cough after returning from a 2-year stay in Liberia. In January 2019, he had been treated in Liberia for malaria, typhoid, and thrush. The patient already had an HIV diagnosis at the time he sought treatment, which we confirmed; he was taking lamivudine/zidovudine/nevirapine (150/300/200 mg/d) combination tablets, trimethoprim/sulfamethoxazole (160/800 mg/d) for pneumocystis prophylaxis, and fluconazole (100 mg/d) for thrush. Despite self-reported perfect compliance with his medication regimen, the patient lost 14 kg body weight and reported worsening fatigue over the 5-month period before he sought care in Virginia. The patient’s social history revealed smoking 30 packs/year and drinking up to 6 beers/day. 

At initial workup, his CD4 T lymphocyte count was 16 cells/mm^3^ and HIV-1 viral RNA was 359 copies/mL. We excluded malaria during differential diagnosis with 3 thin/thick smears. Because the patient exhibited fever and was an immunocompromised returning traveler, we admitted him for further evaluation. Computed tomography (CT) of the chest revealed ground glass opacifications at bases, tree-in-bud nodularity within posterior, lateral, and anterior right upper lobes, and a central necrotic nodule at the left lower lung base measuring 1.3 × 2.1 cm (Appendix Figure). We found associated hilar and aortopulmonic lymphadenopathy measuring up to 8 mm in diameter. He was evaluated by infectious disease clinicians and started on amoxicillin/clavulanic acid (875/125 mg every 12 h) and doxycycline (100 mg every 12 h). We increased fluconazole to 200 mg/d and continued trimethoprim/sulfamethoxazole prophylaxis and antiretroviral therapy. The patient displayed night sweats and fever on days 2 (100.9°F) and 3 (101.5°F). He was afebrile on day 4 and for the remainder of his hospital stay. 

A needle core biopsy of the lung nodule on day 4 revealed necrotizing granulomatous inflammation consisting of epithelioid histiocytes associated with intracellular narrow-based budding yeast (Figure 1, panels B, C) and multinucleated giant cells (Figure 1, panel A). Yeast forms 2–5 μm in size were visible on the hematoxylin and eosin smears. Both histochemical stains for Grocott methenamine silver and periodic acid–Schiff performed on the core biopsy were positive, but a mucicarmine stain was negative. Among the serologic fungal markers tested, serum cryptococcal antigen was negative. The Platelia Aspergillus galactomannan assay (Bio-Rad Laboratories, https://www.bio-rad.com) was elevated at 2.10 (reference range <0.49), and beta-D-glucan (Fungitell; Associates of Cape Cod, https://www.fungitell.com) was negative at 35 pg/mL (reference range <60 pg/mL). The patient was discharged on voriconazole (200 mg 2×/d) in addition to his HIV medication. 

**Figure 1:**
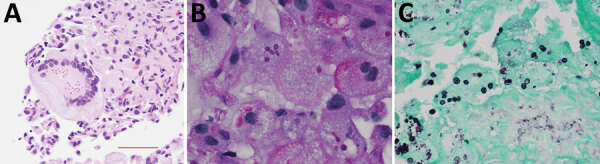
Left lower lobe needle core biopsy histology of *Emergomyces pasteurianus* infection in a patient returning to the United States from Liberia. A) Numerous yeast within a multinucleated giant cell shown by hematoxylin and eosin stain; original magnification ×600. B) Narrow budding yeast shown by periodic acid–Schiff stain; original magnification ×1,000. C) Yeast shown by Grocott methenamine sliver stain; range 2–5 μm.

On day 17, the biopsy culture grew a fungus initially reported on the basis of morphology as presumptive *Emmonsia* sp., which was further identified through sequencing. We saw the patient for follow-up in the clinic on day 31; his appetite had returned, and he had gained 4 kg. We simplified his antiretroviral therapy to bictegravir/emtricitabine and tenofovir alafenamide. Day 37 culture results (LabCorp, https://www.labcorp.com) confirmed *Emergomyces pasteurianus* (formerly *Emmonsia pasteuriana*) through sequencing of internal transcribed spacer regions 1 and 2. MICs for antifungal agents were determined at the University of Texas Health Science Center (San Antonio, Texas, USA) by susceptibility tests at 23°C by broth microdilution (Table 1). 

**Table 1:** MIC/MEC (μg/mL)Antimicrobial susceptibility of antifungal agents for *Emergomyces pasteurianus* isolate from a patient returning to the United States from Liberia and reported cases from the literature*

Case (ref)	AMB	MICA	ANID	FLC	ITC	VOR	POS	ISA	5-FC
This study	0.25	0.03	<0.015	>64	0.06	0.25	0.25	1.0	>64
1 (*8*)	0.031	0.031	0.5	>64	0.125	0.25	0.125	2.0	NA
2 (*9*)	NA	NA	NA	NA	NA	NA	NA	NA	NA
3 (*4*)	1.0	0.05	NA	2.0	0.125	0.25	0.125	NA	NA
4 (*10*)	0.125	0.063	0.0031	>64	0.25	0.25	0.063	1.0	NA
5 (*11*)	NA	NA	NA	NA	NA	NA	NA	NA	NA
6 (*5*)	0.031	<0.008	NA	64	0.063	0.25	0.063	1.0	NA
7 (*5*)	NA	NA	NA	NA	NA	NA	NA	NA	NA
8 (*12*)	NA	NA	NA	NA	NA	NA	NA	NA	NA
9 (*13*)	1.0	0.5†	NA	4.0	0.125	0.25	0.125	NA	NA
10 (*14*)	NA	NA	NA	NA	NA	NA	NA	NA	NA

*Values are MICs (μg/mL), except as indicated. 5-FC, 5-fluorocytosine; AMB, amphotericin B; ANID, anidulafungin; FLC, fluconazole; ISA, isavuconazole; ITC, itraconazole; MICA, micafungin; POS, posaconazole; ref, reference; VOR, voriconazole. †Caspofungin minimal effective concentration.

At day 81 follow-up, the patient reported that his cough had resolved. His CD4 was 54 cells/mm^3^ and viral load was 129 copies/mL. Our plan was to continue prescribing voriconazole for 12 weeks, then repeat the chest CT scan; however, the patient did not return for follow-up. 

The geographic distribution of *E. pasteurianus* is still being described. *Emergomyces* is a dimorphic fungus related to *Emmonsia*, *Histoplasma*, and *Blastomyces* (*1*). This organism is an emerging pathogen among immunocompromised persons, especially those with HIV. *E. pasteurianus* was originally classified within the genus *Emmonsia.* However, the formation of yeast rather than adiaconidia (formerly adiaspores) and the clinical manifestations of emergomycosis suggested that *E. pasteurianus* belongs in a different genus from *Emmonsia* spp. (*1*). Subsequent genetic sequencing supported this relationship (*2*). There is evidence that the number of diagnosed emergomycosis cases are increasing, possibly because of more sensitive diagnostic techniques (*1*). We definitively diagnosed the infection in this patient through fungal cultures developed from lung biopsy samples, in which the organism readily grew as a filamentous fungi on Sabouraud dextrose agar, Mycocel agar, and brain–heart infusion agar at 25˚C. Colonies on Sabouraud dextrose agar incubated at 25˚C appeared white and compact and became domed/heaped over time (Figure 2, panel A). The reverse side started as white/cream and progressed to tan. 

**Figure 2:**
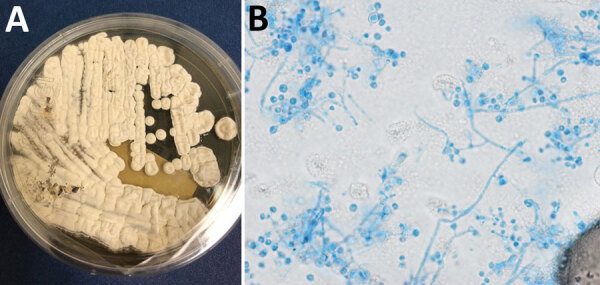
Lung nodule biopsy and fungal culture isolate of *Emergomyces pasteurianus* infection in a patient returning to the United States from Liberia. A) Colony morphology on Sabouraud dextrose agar at 14 days. B) Lactophenol cotton blue tape prep; original magnification ×1,000.

The microscopic appearance of the mold form of *E. pasteurianus* has been described as septate, hyaline hyphae, with short conidiophores arising at right angles that may show a slight swelling at the tip and typically produce >1 round conidia on short thin denticles. Conidia may also appear directly off the hyphae. The conidia are described as hyaline, thin-walled, 2–4 µm in size (*2*,*3*), which mirrors our experience. The yeast form (grown at 37˚C or present in tissue) is described as 2–5 µm with narrow budding. Bipolar budding and formation of giant cells with broad-based budding have also been reported (*3*–*5*); however, we did not definitively observe those forms in this case (Figure 2, panel B). 

Clinical manifestations of emergomycosis may include dyspnea, pleuritic chest pain, and pink to purple nodular skin lesions (*4*). One study reported rapid progression of respiratory failure and death (*5*). Skin rash has been reported in some cases, but frequency of this clinical sign is unknown. CT imaging may show necrotizing cavitary lesions (*4*) or diffuse pulmonary infiltrates (*5*). Histopathology of skin lesions have shown yeast forms (*4*). Risk factors for emergomycosis include HIV (CD4 count <10 cells/mm^3^) (*4*), B-cell chronic lymphocytic leukemia with neutropenia, and chronic prednisone therapy (*5*). Chronic kidney disease was present in 2 case-patients, but association was uncertain (*5*). 

Data are limited on antifungal susceptibilities for *E. pasteurianus*. The organism appears to have low MICs for itraconazole, posaconazole, and voriconazole but higher MICs for fluconazole and flucytosine (*4*). Although echinocandins generally have low MICs for the mold form of *Emergomyces*, activity in the pathogenic yeast form is less well known and significant discrepancies have been noted in other dimorphic fungi (*6*). A study of 50 clinical isolates of *E. africanus* demonstrated consistently low MICs to voriconazole, posaconazole, and itraconazole for both yeast and mold forms, with consistently elevated MICs for echinocandins and fluconazole (*7*). Although no guidelines exist to direct treatment for emergomycosis, multiple treatment courses have been used with varying outcomes (Table 2). 

**Table 2:** Reported cases of *Emergomyces pasteurianus* (formerly *Emmonsia pasteuriana*) from the literature*

Case (ref)	**Year**	**Location**	**Patient age, y/sex**	**Patient medical history**	**Clinical features**	**Specimen cultured**	**Treatment**	**Outcome**
1 (*8*)	1998	Italy	40/F	HIV/AIDS	Skin ulcerations, weight loss	Skin biopsy	Amphotericin	Died from unrelated cause
2 (*9*)	2008	Spain	46/M	HIV (CD4 134 cells/μL, HCV, liver transplant	Nodular skin lesions (ulcerating), bilateral pulmonary infiltrates, liver failure	Skin biopsy	Liposomal amphotericin B (2 wk), decreased immunosuppression	Died
3 (*4*)	2012	India (Nepal native)	38/F	HIV (CD4 <10 cells/μL)	Nodular skin lesions, weight loss, dyspnea, bilateral pulmonary infiltrates (LUL necrotizing lesion),	Pulmonary nodule biopsy	HAART, 2 wk, amphotericin B, then itraconazole, 12 mo	Survived
4 (*10*)	2015	China	43/M	Renal transplant	Nodular skin lesions (painful, ulcerating), bilateral pulmonary nodules, fungitell negative at first then up to 339 pg/mL	Skin biopsy	Amphotericin B, voriconazole, and caspofungin, 2 wk (ongoing)	Survived
5 (*11*)	2015	China	30/F	CMV enteritis, urticaria (on prednisone), HIV negative	Nodular skin lesions	Skin biopsy	Oral voriconazole, 2 mo	Survived
6 (*5*)	2016	Netherlands (Moroccan ancestry)	62/F	B cell non-Hodgkin lymphoma, cirrhosis, CKD, T2DM, AIHA; 50 mg/d prednisone	Nodular skin lesions, dyspnea, RUL nodule	Skin biopsy	Posaconazole, decreased steroid dosing, 14 mo	Survived
7 (*5*)	2017	Netherlands (Iraqi nationality)	80/M	B-CLL, CKD	Encephalopathy, fever, respiratory failure, sepsis	BAL	Amphotericin B	Died
8 (*12*)	2019	Uganda	38/F	HIV (CD4 140 cells/μL)	Nodular skin lesions	Skin biopsy	Fluconazole, 6 wk, with clinical worsening followed by itraconazole, 8 wk	Survived
9 (*13*)	2020	India	27/F	HIV/AIDS	Weight loss, cough, skin lesions	Skin biopsy	Amphotericin, 2 wk, itraconazole, 12 mo	Survived
10 (*14*)	2020	Hong Kong	61/M	Renal transplant	Pneumonia	Lung biopsy	Amphotericin, 8 wk voriconazole, 10 wk	Died

*AIHA, autoimmune hemolytic anemia; B-CLL, B cell chronic lymphocytic lymphoma; CKD, chronic kidney disease; CMV, cytomegalovirus; HAART, highly active antiretroviral therapy; HCV, hepatitis C virus; LUL, left upper lobe; RUL, right upper lobe; T2DM, type 2 diabetes mellitus; ref, reference.

Serologic markers have been shown insufficient for diagnosing *E. pasteurianus* infection. The galactomannan assay was positive in the only previous case reporting a result and in our case (*5*). Beta-d-glucan testing was negative in our patient but was reported positive in 1/4 cases in other studies (*8*). Cross-reactivity with the histoplasma urine antigen has been reported (*8*). However, none of those tests are specific for *Emergomyces*, and they have not been systematically studied as markers for this specific pathogen. 

## Conclusions

Our study provides evidence of possible *E. pasteurianus* endemicity in Liberia and adds to the literature on susceptibilities for this emerging pathogen. We provide further evidence of low MICs to newer generation triazoles, suggesting their utility in empiric therapy, but additional data is needed to clarify formal breakpoints. Given the gaps in our knowledge about *E. pasteurianus*, public health providers should be aware of clinical manifestations of emergomycosis and consider it in the differential diagnosis, especially in regions where its presence is known. 

Appendix Additional information about study of *Emergomyces pasteurianus* in patient returning to the United States from Liberia.
