# Supplementary material for: Emergomyces pasteurianus in Man Returning to the United States from Liberia and Review of the Literature
Source: Emerg Infect Dis. 2023 Mar;29(3):635–9. doi: 10.3201/eid2903.221683 (PMC9973675; doi:10.3201/eid2903.221683)
Supplement: Appendix — Additional information about study of Emergomyces pasteurianus in patient returning to the United States from Liberia. [file 22-1683-Techapp-s1.pdf]

Article DOI: https://doi.org/10.3201/eid2903.221683
EID cannot ensure accessibility for supplementary materials supplied by authors. Readers who have difficulty accessing supplementary content should contact the authors for assistance.

Emergomyces pasteurianus in Man Returning to the United States from Liberia and Review of the Literature

Appendix

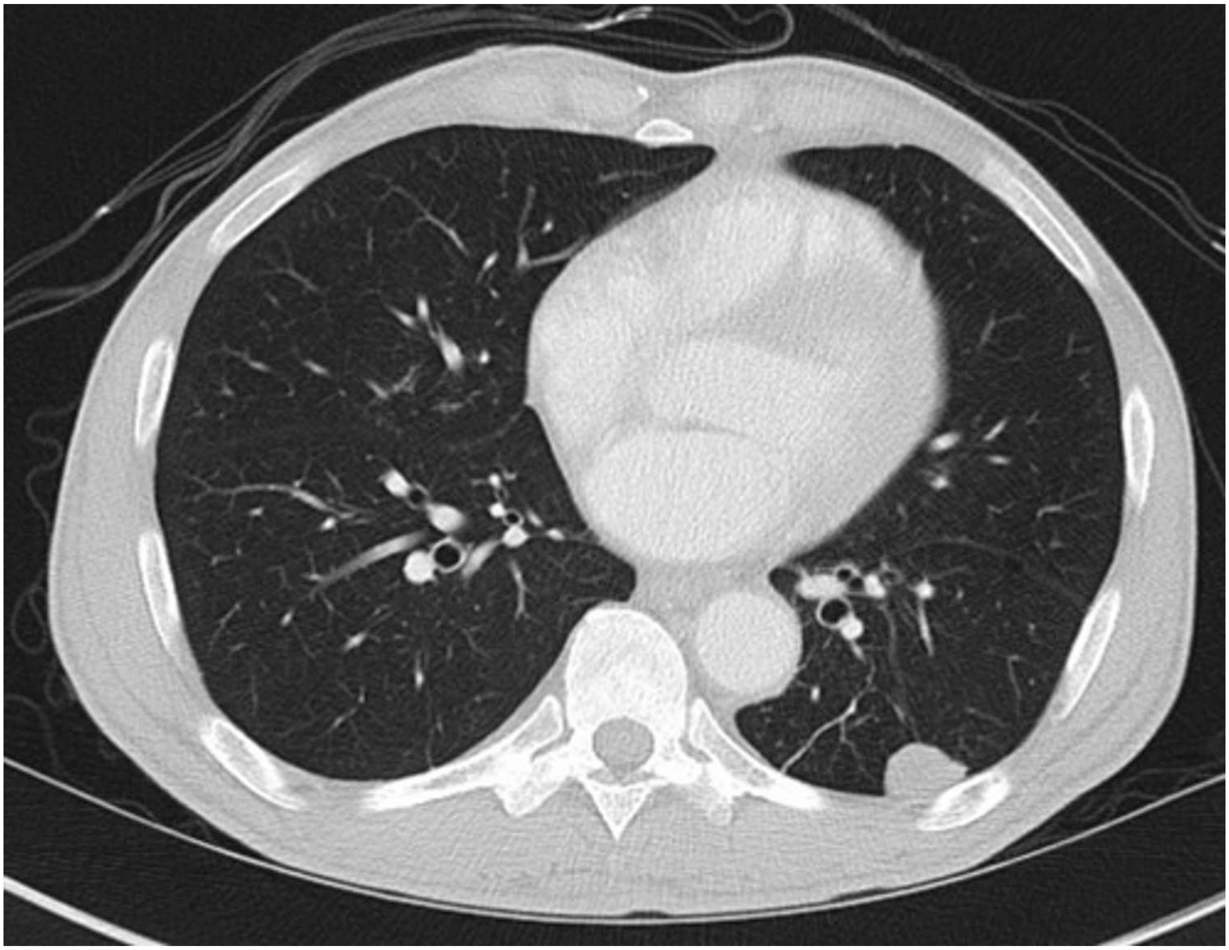

Appendix Figure. Computed tomography scan: lung nodule is apparent in left lower lobe with evidence of central necrosis
